# Meeting Report: Methylmercury in Marine Ecosystems—From Sources to Seafood Consumers

**DOI:** 10.1289/ehp.11211

**Published:** 2008-07-23

**Authors:** Celia Y. Chen, Nancy Serrell, David C. Evers, Bethany J. Fleishman, Kathleen F. Lambert, Jeri Weiss, Robert P. Mason, Michael S. Bank

**Affiliations:** 1 Department of Biological Sciences; 2 Dartmouth Toxic Metals Research Program, Dartmouth College, Hanover, New Hampshire, USA; 3 BioDiversity Research Institute, Gorham, Maine, USA; 4 Ecologic: Analysis and Communications, Woodstock, Vermont, USA; 5 U.S. Environmental Protection Agency, Boston, Massachusetts, USA; 6 Department of Marine Sciences, University of Connecticut, Avery Point, Connecticut, USA; 7 Department of Environmental Health, Harvard School of Public Health, Boston, Massachusetts, USA

**Keywords:** bioaccumulation, human health, mercury biomonitoring, mercury exposure, methylmercury

## Abstract

Mercury and other contaminants in coastal and open-ocean ecosystems are an issue of great concern globally and in the United States, where consumption of marine fish and shellfish is a major route of human exposure to methylmercury (MeHg). A recent National Institute of Environmental Health Sciences–Superfund Basic Research Program workshop titled “Fate and Bioavailability of Mercury in Aquatic Ecosystems and Effects on Human Exposure,” convened by the Dartmouth Toxic Metals Research Program on 15–16 November 2006 in Durham, New Hampshire, brought together human health experts, marine scientists, and ecotoxicologists to encourage cross-disciplinary discussion between ecosystem and human health scientists and to articulate research and monitoring priorities to better understand how marine food webs have become contaminated with MeHg. Although human health effects of Hg contamination were a major theme, the workshop also explored effects on marine biota. The workgroup focused on three major topics: *a*) the biogeochemical cycling of Hg in marine ecosystems, *b*) the trophic transfer and bioaccumulation of MeHg in marine food webs, and *c*) human exposure to Hg from marine fish and shellfish consumption. The group concluded that current understanding of Hg in marine ecosystems across a range of habitats, chemical conditions, and ocean basins is severely data limited. An integrated research and monitoring program is needed to link the processes and mechanisms of MeHg production, bioaccumulation, and transfer with MeHg exposure in humans.

Methylmercury (MeHg), a toxic organic form of mercury, is the predominant form found in fish tissue. People who consume large amounts of fish contaminated with MeHg have a higher body burden than do people who do not consume fish (Mahaffey and Mergler 1998). The developing human nervous system is a sensitive target for MeHg exposure, putting developing fetuses and young children at the highest risk for harm [Clarkson et al. 2003; Grandjean et al. 2005; National Academy of Sciences Committee on the Toxicological Effects of Methylmercury (NAS) 2000]. Marine apex predators such as sharks, seabirds, and marine mammals are also at risk from elevated MeHg exposure (Braune et al. 2006; Garcia-Hernandez et al. 2007; Kemper et al. 1994).

To date, research on the environmental fate of Hg and MeHg has focused primarily on freshwater systems and upland watersheds. Far less research has been conducted in marine environments where the transport and migration of water, contaminants, and fisheries resources make the identification of sources and receptors of Hg and MeHg contamination particularly challenging (Knap et al. 2002). However, because the main vector for MeHg exposure in humans in the United States is fish and shellfish consumption, 60% of which is derived from marine systems (Sunderland 2007), the links between MeHg bioaccumulation in estuarine, coastal, and open-ocean ecosystems and human exposure need to be better understood.

The existing research in marine systems has focused largely on Hg biogeochemistry in the open ocean (Benoit et al. 2003; Fitzgerald et al. 2007; Hammerschmidt and Fitzgerald 2004; Laurier et al. 2004; Lawrence et al. 1999; Mason and Gill 2005; Mason et al. 1999; Mason and Lawrence 1999). A limited number of extensive studies have been conducted in specific coastal systems, such as Long Island Sound, Scheldt River Estuary, and Chesapeake Bay (Baeyens et al. 2003; Hammerschmidt and Fitzgerald 2006; Mason et al. 2006a, 2006b). Early studies on health effects focused on acute exposures in Minimata, Japan, and later extensive studies investigated chronic low-level exposures in Seychelles and Faroe Islands (NAS 2000); these exposures resulted from consumption of marine organisms (the Seychelles population consumed marine fish, and the Faroe Islands population consumed pilot whale). Fetal exposures to Hg in both these populations have been associated with neurologic deficits in children, in some cases many years after exposure (Mergler et al. 2007). Although the breadth of Hg research is great, the links between marine sources and ultimate human exposure have not been made across large geographic areas. There is now a need to adopt a systems approach to the study of MeHg in marine ecosystems with more focus on the processes controlling the transfer of MeHg in marine food webs, particularly those that link MeHg sources to seafood consumed by humans.

With this in mind, the Dartmouth Superfund Basic Research Program, with support from the National Institute of Environmental Health Sciences and New Hampshire Sea Grant, convened a workshop titled “Fate and Bioavailability of Mercury in Aquatic Ecosystems and Effects on Human Exposure” in November 2006. The goal of this meeting was to bring together ecosystem scientists and human health scientists for cross-disciplinary discussions to identify research and monitoring priorities linking the fate and bioaccumulation of Hg in the marine environment to human exposure. Here, we summarize the major research and monitoring needs identified for each of the three workshop themes: *a*) the biogeochemical cycling of Hg in marine ecosystems, *b*) the trophic transfer and bioaccumulation of MeHg in marine food webs, and *c*) human exposure to Hg from marine fish and shellfish consumption. This article is not intended to present an exhaustive review of the state of the science; several review papers on Hg in marine ecosystems that have been published previously are listed in Table 1.

## Biogeochemical Cycling of Hg in Marine Ecosystems

Research and monitoring on the biogeochemical cycling of Hg in marine ecosystems are critical to expanding current understanding of the MeHg sources that contaminate marine fish. Important insights can be gained from the extensive research in freshwater ecosystems, but biogeochemical processes in freshwater and saltwater likely differ. For example, organic matter decreases from watersheds to the open ocean, and sulfate concentrations increase; both constituents are known to strongly influence Hg bioavailability to sulfur-reducing bacteria (Benoit et al. 2003; Sunderland et al. 2006). They also affect the flux of Hg and MeHg from sediments to the water column. Freshwater studies and the existing literature on marine Hg cycling (Table 1) need to be extended to investigate the factors controlling MeHg production, sediment flux, and bio-transfer in marine ecosystems. The workgroup identified three questions that should be addressed: *a*) Where is Hg methylation occurring in the ocean? *b*) How is MeHg mobilized from sediments to water in coastal ecosystems? *c*) What is the relative importance of benthic biotransfer of Hg into aquatic food webs.

### Research questions

#### Where is Hg methylation occurring in the ocean?

Methylation is a key process in the transformation of inorganic Hg to the MeHg that bioaccumulates in food webs. Although Hg methylation has been studied in depth in coastal and open-ocean environments, studies of tropical and polar regions and deep ocean basin are limited.

There are three potential regions of methylation in marine ecosystems—coastal and slope sediments, low-oxygen waters below productive ocean waters, and deep ocean sediments (Kraepiel et al. 2003). Current research suggests that net MeHg production in coastal marine sediments is one of the more important sources and thus a potential source for MeHg in marine fish (Hammerschmidt and Fitzgerald 2004; Sunderland et al. 2006). Measurements of methylation sources in a wider range of marine conditions are needed to evaluate the relative importance of coastal marine sediments to MeHg concentration in shellfish, fish, and other biota.

Methylation in coastal sediments is largely controlled by bacterial activity and the bio-availability of inorganic Hg, which is highly dependent on sediment and porewater concentrations of organic carbon and sulfide. Some, but not all, sulfate-reducing bacteria are thought to be the primary methylators of Hg in sediment (Benoit et al. 2003). However, recent evidence suggests that other bacteria, including iron reducers, methylate Hg as well (Slowey and Brown 2007). Demethylation appears to be influenced by both abiotic (photochemical) and biotic processes, but less is known about these factors. The controls on both methylation and demethylation in marine systems should be a more active area of research.

These patterns would be most effectively examined within the context of total Hg and MeHg concentrations in the water column. However, concentrations of MeHg in most of the oceans, except for coastal oceans and the equatorial Pacific, are low and difficult to measure (Table 2) (Fitzgerald et al. 2007). For example, recent model results support the idea that the concentrations of total Hg and MeHg differ between ocean basins, as does the rate of change in concentrations (Laurier et al. 2004; Strode et al. 2007; Sunderland and Mason 2007), but more study and data are required to confirm these trends.

#### How is MeHg mobilized from sediments to water in coastal ecosystems?

Although the main source of MeHg in coastal ecosystems is known to be microbial methylation in sediments, few studies have examined the rates of mobilization or diffusion of that MeHg from the source to the water column where it is available to the food web (e.g., Choe et al. 2004; Covelli et al. 1999; Mason et al. 2006b). Although biogeochemical factors such as organic carbon, oxygen, and sulfide concentrations appear important, their combined influences on methylation, demethylation, and MeHg mobilization in marine systems are poorly understood. Existing studies suggest that their influence depends on factors that influence partitioning of MeHg to the solid phase, such as organic carbon and solid sulfide content, and sediment redox status (Gill et al. 1999; Mason et al. 2006b), but the relationships between biogeochemical conditions and the mobilization of MeHg into marine waters need to be clarified.

Determination of MeHg flux is constrained by the limitations of current methods, which can vary—depending on the method—by an order of magnitude or more. There is a need for *in situ* measurement devices that do not hinder advective processes (e.g., diffusive gel time series) (Merritt and Amirbahman 2007) and for the development of methods and measurements that work across ranges of biogeochemical conditions.

#### What is the relative importance of benthic biotransfer of Hg into aquatic food webs?

The role of benthic biota in transferring Hg to the higher-trophic-level fish and shellfish species consumed by humans is poorly understood. Existing work suggests that bioturbation of sediments by benthic infauna can affect methylation rates and distribution of MeHg in sediment (Benoit et al. 2006). Benthic fauna in Hg-contaminated sediments have also been shown to exhibit higher Hg concentrations than those in more pristine sites, suggesting that biotic transfer from this food pathway may contribute to elevated total Hg levels in high-trophic-level organisms (Chen CY, Dionne M, Jackson BP, unpublished data). However, organic content of sediments diminishes the bioavailability of MeHg to benthic fauna, which may result in lower levels of bio-transfer from highly organic-rich sediments (Lawrence and Mason 2001; Mason and Lawrence 1999). The factors controlling bio-transfer of MeHg by benthic fauna need to be identified.

The relative importance of benthic fauna in biotransfer should also be more closely examined. Recent studies in freshwater and marine systems indicate that MeHg concentrations are higher in pelagic than in benthic fauna, suggesting that chemical flux into the water column may be more important than biotransfer mechanisms (Chen CY, Dionne M, Jackson BP, unpublished data; Gorski et al. 2003; Power et al. 2002), but this has not been extensively investigated.

### Monitoring needs

To identify the important factors controlling methylation, monitoring data should be collected to characterize the spatial and vertical distribution of Hg and MeHg in ocean waters and sediments across a range of marine ecosystems. This range should include coastal margins, where riverine inputs of MeHg may be important, to the open ocean and the deep ocean, where sources of dimethylmercury are present.

Better analytical techniques are needed to improve detection limits of Hg and MeHg in marine waters, given that levels in most of the world’s oceans are difficult to measure. In addition, measures of ancillary variables in water and sediments are needed to identify the factors controlling Hg methylation and demethylation [e.g., selenium, iron, manganese, sulfide, dissolved organic carbon (DOC), pH, chloride, productivity, and nutrients].

Standardized measurements of methylation rates and MeHg flux from sediments across a range of ecosystem types would aid in validating existing MeHg model results and inform a better understanding of the magnitude of chemical flux of MeHg from sediments. These methylation rates and MeHg fluxes should be linked to measurements of Hg and MeHg in benthic infauna and epifauna to quantify relative contributions of chemical and biotic flux of MeHg to the water column.

## Trophic Transfer and Bioaccumulation of MeHg in Marine Food Webs

Trophic transfer and bioaccumulation of MeHg in marine food webs link MeHg production to MeHg exposure in humans and wildlife. Although trophic transfer and effects of MeHg in freshwater food webs have been well characterized in North America (Driscoll et al. 2007; Evers and Clair 2005; Watras et al. 1998), much more attention is needed on marine ecosystems. The workgroup identified three priority research questions: *a*) What is the key entry point for MeHg in the base of the food web in marine ecosystems? *b*) What are the factors influencing the transfer of MeHg from the base of the food web to higher-trophic-level organisms consumed by humans? *c*) what types of MeHg impacts have been measured in marine biota, and which organisms could serve as useful indicators for monitoring MeHg spatiotemporal trends in marine ecosystems?

### Research questions

#### What is the key entry point for MeHg into the base of the food web in marine ecosystems?

Studies of inland aquatic ecosystems have found the greatest degree of MeHg bioaccumulation in the food web to occur between concentrations in water and concentrations in phytoplankton. For example, the concentration of MeHg has been shown to increase by up to five orders of magnitude, with the percentage of total Hg as MeHg increasing an average of 1% in water to 10% in phytoplankton (Driscoll et al. 2007; Fitzgerald et al. 2007).

Although estimates of bioconcentration exist for coastal waters, little is known of the bioconcentration by phytoplankton in the open ocean (Fitzgerald et al. 2007). In freshwater studies, MeHg concentrations in water do not consistently predict concentrations at the base of the food web. Limnologic factors such as pH, DOC, and nutrients can have important effects on the bioaccumulation of MeHg by phytoplankton and zooplankton in these ecosystems (Chen et al. 2005; Driscoll et al. 2007; Pickhardt et al. 2002; Watras et al. 1998). In marine ecosystems, the chemical speciation of MeHg and its bioavailability are influenced greatly by the abundance of chlorine and sulfur, but less so by DOC and variation in pH (Lawson and Mason 1998). The presence of Hg or MeHg as inorganic or organic complexes determines the passive or active uptake by algal cells (Mason 2002). However, the degree to which quality and quantity of DOC affect Hg bioaccumulation is poorly understood. More data are needed to characterize bioaccumulation processes in phytoplankton across a range of marine ecosystems.

#### What are the factors influencing the transfer of MeHg from the base of the food web to higher-trophic-level organisms consumed by humans?

Existing studies of freshwater and marine food webs show increasing MeHg concentrations with increasing trophic position as measured by stable isotopes (Bank et al. 2007; Driscoll et al. 2007; Hammerschmidt and Fitzgerald 2006). Both freshwater and marine fish also appear to have higher MeHg concentrations with increasing size and age (Hammerschmidt and Fitzgerald 2006; Wiener and Spry 1996). However, other aspects of trophic transfer of MeHg are far more difficult to track in marine food webs. Species-specific life-history characteristics, migration patterns, ontogenetic shifts in diet, and differences in life span are poorly known for most marine species (Bank et al. 2007; Hammerschmidt and Fitzgerald 2006). In addition to the need to characterize MeHg concentrations in a range of species across trophic levels, ages, and habitats, a better understanding of the general ecology of marine species is needed to properly interpret differences in MeHg burden between and within species across space and time.

Food web characteristics influence the transfer of MeHg from its sources to higher trophic levels. Humans consume fish from both demersal and pelagic fisheries, but little is known about the relative degree of MeHg bioaccumulation and trophic transfer in these two food webs. Some evidence suggests that MeHg burdens in similar trophic-level fish are higher in demersal than in pelagic species (Garcia-Hernandez et al. 2007). The influence of different food sources and food webs on MeHg bioaccumulation in fish species needs to be characterized, particularly for species most consumed by humans.

Food sources and food webs also influence the bioaccumulation of MeHg in apex predators such as marine mammals and birds. Marine mammals that have among the highest MeHg body burdens include toothed cetaceans and pinnipeds that feed on fish (Kemper et al. 1994; Thompson 1996; Wagemann et al. 1998). In contrast, baleen cetaceans have lower Hg levels, likely due to their diet of plankton (Hobson et al. 2004). Studies of seabirds suggest that habitat type and functional feeding group may influence MeHg bioaccumulation rates in higher-trophic-level organisms. For example, MeHg bioaccumulation rates differ between benthic- and pelagic-feeding birds and between inshore and offshore species (Goodale et al., in press; Thompson et al. 1998). More data are needed to determine whether the influences of habitat type, feeding strategy, and diet on MeHg bio-accumulation are consistent across a range of ocean ecosystems and taxonomic groups.

#### What types of MeHg impacts have been measured in marine biota, and which organisms could serve as useful indicators for monitoring MeHg spatiotemporal trends in marine ecosystems?

Elevated environmental Hg concentrations have been widely documented in marine biota and extreme levels are regularly reported (Bustamante et al. 2003; Kim et al. 1996). The direct effects of elevated MeHg on marine biota can include impacts on neurologic end points and memory, locomotion, and cognition, as well as changes in brain neurochemical receptor density (Basu et al. 2005; Scheuhammer et al. 2008). Adverse effects may further manifest as immunosuppression, which may make individuals more susceptible to disease, as has been measured in cetaceans and pinnipeds (Gauthier et al. 1998; Lalancette et al. 2003). Direct reproductive effects associated with high Hg levels have been documented in bird species in freshwater ecosystems (Burgess and Meyer 2008; Evers et al. 2008), and Braune et al. (2006) documented egg Hg concentrations in the ivory gull that exceed twice the adverse effect threshold for eggs in the common loon (Evers et al. 2003), suggesting that seabirds may be experiencing similar reproductive effects as freshwater birds. More research is needed to understand both the mechanisms and thresholds for adverse neurologic, immunosuppressive, and reproductive effects in marine organisms.

The interpretation of Hg effects data in marine biota is complicated by the fact that sensitivity to MeHg toxicity can vary among taxa and foraging guilds. For example, Heinz et al. (2008) found eggs of marine birds to be less sensitive to dosing with MeHg than those of terrestrial species. Moreover, information is needed about the interactions of MeHg with other chemicals and contaminants, including the ability of selenium to both reduce and enhance MeHg toxicity (Heinz G, personal communication). Finally, in addition to the impacts of elevated MeHg on individuals within a species, more research is needed relating Hg concentrations to population-level effects in highly exposed species.

### Monitoring needs

Detailed information on MeHg concentrations in marine food webs across estuary, coastal, and open-ocean habitats is needed to better understand effects and to monitor changes in environmental MeHg loads over space and time. Emphasis should be placed on capturing a range of productivity from oligotrophic to eutrophic aquatic systems, and MeHg measurements should be conducted across a broad range of indicator taxa. Selected species should represent differing foraging guilds, habitats, and geographic areas and be prioritized based on *a*) existing Hg data, *b*) commercially harvested species for human consumption, *c*) sensitivity to MeHg, and *d*) degree of conservation concern. Among chosen indicator taxa, emphasis should be placed on measurements of relevant tissue types to best relate MeHg concentrations to specific neurologic, behavioral, and reproductive effects in marine biota (Wolfe et al. 2007). Monitoring efforts should also include stable isotope measurements, such as change in ^13^C and ^15^N ratios, in lower- and upper-trophic-level taxa in order to detect shifts in trophic structure and position (Hobson 1993; Hobson et al. 1994).

In order to capture ecologically meaningful changes in MeHg concentrations, monitoring should be conducted in both low- and high-trophic-position organisms. To understand the entry of MeHg at the base of the food web, monitoring should include measurements of MeHg in phytoplankton, zooplankton, and benthic invertebrates. At the top of the food web, five broad groups of apex predators representing some of the highest MeHg concentrations in marine organisms may be useful indicators: sharks, estuarine birds, seabirds, pinnipeds, and toothed whales.

Studies on these apex predator groups have been conducted in the north temperate Atlantic Ocean (Spalding et al. 2007), suggesting that monitoring these taxa is feasible. But regional data for Hg bioaccumulation in apex species from some marine waters (e.g., the northeast United States) remain limited for sharks, pinnipeds, and toothed whales (Gaskin et al. 1973, 1979; Lake et al. 1995), estuarine birds (Cohen et al. 2000; Custer and Mulhern 1983; Rattner et al. 2000; Shriver et al. 2006), and seabirds (Burger 2002; Burger and Gochfeld 1995, 2003, 2004; Gochfeld 1980; Gochfeld and Burger 1998; Gochfeld et al. 1996; Goodale et al., in press). Moreover, the great variability in units of measure, species chosen, age and sex class, and tissue type point to the need for more standardized Hg monitoring protocols for marine biota.

## Human Exposure to Hg from Marine Fish and Shellfish Consumption

Considerable research has been conducted on the human health implications of exposure to MeHg (Clarkson et al. 2003; Clarkson and Magos 2006; Grandjean et al. 2005; Mergler et al. 2007; NAS 2000). In the United States, most people receive their highest Hg exposure through consumption of seafood (Sunderland 2007). However, the relationship between the ecosystem fate of MeHg in freshwater and marine systems is poorly understood, and little is known about the effects of ecosystem variability on human exposure. In addition to Hg, seafood is a potential source of other contaminants, as well as a source of important nutrients such as omega-3 oils. There is a research need for well-defined and meaningful data supporting parameters of risk and benefit. Monitoring needs include increased tracking of the sources of the fish people eat. The workgroup identified three research questions: *a*) What is the cumulative risk of MeHg and other cocontaminants in fish [polychlorinated biphenyls (PCBs), dioxin, etc.], and what are the trade-offs in benefits from eating fish? *b*) What are the patterns of Hg exposure and consumption for the most highly exposed human populations? *c*) What are peoples’ responses to risk–benefit messages, and how can they be improved?

### Research questions

#### What is the cumulative risk of MeHg and other cocontaminants in fish (PCBs, dioxin, etc.), and what are the trade-offs in benefits from eating fish?

Research on fish contamination and human exposure is often framed in terms of a single contaminant. This approach may not be appropriately holistic for protecting human health. For public policies on fish to be protective of public health, there is a need to define an appropriate baseline for cumulative exposure to MeHg and other contaminants and a means of determining how regulation might alter the combined exposure levels. Developing species-specific pollution matrices in conjunction with fishery sustainability data may assist in identifying those species and fish populations that pose the greatest threat from both human and environmental health standpoints. These matrices will need to be sensitive to variation in species contamination across time and geographies.

In addition, there is a need for investigating the risks and benefits of fish consumption (Budtz-Jørgensen et al. 2007; Stern 2005) in studies that separate and clarify opposite impacts on health outcomes. This has been an area of growing scientific discussion. Studies such as Oken et al. (2005) have found higher fish consumption in pregnancy to be associated with better infant cognition, and higher Hg levels to be associated with lower cognition. These findings and others (Domingo et al. 2007) have led many health professionals and organizations such as the American Heart Association to recommend consumption of fish with high omega-3s and low MeHg. However, there is a need to better understand the cumulative effects of the other nutrients and contaminants in fish. Investigating these effects, independently or interactively, in species most commonly preferred by humans is a priority. Research on human health and fish consumption needs to reflect the variability of individual exposure over time and space.

#### What are the patterns of Hg exposure and consumption for the most highly exposed human populations?

There are numerous factors that affect MeHg exposure levels in individuals or subpopulations, such as ethnic differences in fish consumption preferences, diet, genetic differences, age, and uptake–excretion variation (Canuel et al. 2006). To date, analysis of the National Health and Nutrition Examination Survey data set shows that Asians, Native Americans, and Pacific Island populations have higher Hg concentrations in their blood (Hightower et al. 2006). In addition, studies show coastal populations having higher Hg concentrations than inland populations (Crépet et al. 2005; Denger et al. 1994; McKelvey et al. 2007). It is important to develop effective tools for capturing these variations and their effects, both for bolus and general exposures. There is also a need for a clinical definition for the subtle symptoms of concern associated with fish consumption resulting in chronic low-level exposures to MeHg that do not fit the current definition for “mercury poisoning” (Hightower and Moore 2003).

Detailed information is needed about the consumption patterns, fish and shellfish species preferences, and regional sources of fish for those who have been shown to carry the highest levels of MeHg in their bodies. In addition, for these highly susceptible individuals, the per capita exposure should be scaled to the species of fish consumed. Individual variation in per capita Hg intake suggests that geography counts where Hg exposure is concerned (Burger et al. 2005). To control exposure, there is a need for research that identifies whether geographic supply regions, fishing methods, and country of origin affect variability in Hg concentrations in the fish available to consumers in different areas of the United States.

#### What are people’s responses to risk–benefit messages, and how can they be improved?

Challenges still remain with regard to balancing risk–benefit messages regarding fish consumption and Hg, and there is a great need to study how people respond to different messages on fish consumption advisories, especially those intended for pregnant women (Knuth et al. 2003; Oken et al. 2003). Some investigators argue that fish consumption advisories are unbalanced in their focus on the health risks of fish consumption without informing the public about the health benefits of consuming appropriate amounts of low-Hg fish (Arnold et al. 2005). However, some advocacy groups contend that consumption advisories do not go far enough in warning vulnerable populations about the risks of eating the most popular forms of fish. High-risk populations, who heavily consume fish and foods originating from the ocean, may require more intensive education efforts to ensure that species-specific and local water-body consumption advisory information is available (Arnold et al. 2005).

### Monitoring needs

Hg monitoring efforts for human exposure should focus on highly exposed populations, commonly consumed fish, and areas of high commercial seafood production. Commonly consumed fish may require a tracking system, similar to beef, to identify catch locations and sources of highly contaminated fish. Such a tracking system should include information on catch location, distribution chain from source areas to consumers, type of fish and brand of the fish product, and assessments of MeHg levels in fish from different regions. There appear to be significant regional differences in Hg concentration by species that are not captured in the Food and Drug Administration’s national database (Burger et al. 2005; Sunderland 2007). Commercial and noncommercial sources (i.e., recreational fish) will likely need to be evaluated separately.

Information on species-specific MeHg concentrations, frequency data, and amount of consumption will be required for human exposure studies to be informative (Burger et al. 2005; Mergler et al. 2007; Sunderland 2007). In addition, special monitoring may be required to evaluate MeHg levels in uncommon fish species that are regularly consumed by certain ethnic sectors of the public. In order to address the problem of multiple exposures to contaminants via fish consumption, measurement of other contaminants (e.g., PCBs, dioxin, polycyclic aromatic hydrocarbons) could also be monitored in commercially harvested species as has been done in some existing monitoring programs (e.g., U.S. Environmental Protection Agency National Coastal Assessment and Gulfwatch Programs).

Consumption data suggest that a common route of MeHg exposure for most Americans is canned tuna (Sunderland 2007). There is known variation between white (albacore tuna) and light (skipjack tuna) varieties, the former having MeHg concentrations > 3 times higher than the latter (Burger and Gochfield 2004; Sunderland 2007). Better monitoring of the national distribution system for canned tuna is needed (type, brand, variation, region, etc.). Data on the sources, species, MeHg concentrations, and markets for canned tuna are necessary to evaluate an important source of MeHg exposure to humans.

## Summary

Hg research in marine ecosystems is a growing field that holds great promise for improving our understanding of the critical linkages among the sources of MeHg in marine systems, the processes that govern bioaccumulation in higher-trophic-level organisms, and human exposure to MeHg through seafood consumption. To realize this potential, however, research and monitoring efforts must be coordinated in a way that helps answer targeted scientific and policy-relevant questions. Based on the deliberations of the 43 participants in the Dartmouth workshop, this report identifies several key research questions and recommended monitoring approaches that should guide future interdisciplinary work. We suggest that the study of Hg in marine ecosystems where humans have the greatest potential to be exposed is severely data limited and that research and monitoring efforts should be expanded considerably. Moreover, we suggest that research and monitoring initiatives should take an integrated approach that addresses the poorly understood linkages among marine sources, biotransfer processes, and bioaccumulation mechanisms that put humans at risk of exposure to MeHg. Within this integrated approach, there is an overarching need to collect data and information across a range of habitats, chemical conditions, and ocean basins and to relate the resulting spatial patterns of MeHg bioaccumulation to the food sources of at-risk human and wildlife populations. Finally, we suggest that to advance these recommendations, an organized Hg monitoring effort in marine systems should be developed to characterize the spatial and temporal variability of MeHg in various compartments much like the proposed Hg monitoring network for freshwater and upland systems (Mason et al. 2005). Together, these recommendations will help elucidate the patterns and processes influencing the transfer of MeHg from marine sources to human exposure.

## Figures and Tables

**Table 1:** Selected reviews of Hg biogeochemistry, trophic transfer, biomonitoring, and human health.

Topic	Key references
Hg biogeochemistry	Fitzgerald and Clarkson 1991
	Fitzgerald et al. 1998
	Morel et al. 1998
	Ullrich et al. 2001
	Mason and Benoit 2003
	Fitzgerald et al. 2007
Hg trophic transfer	Mason et al. 1995
	Mason 2002
	Wiener et al. 2003
	Mason and Benoit 2003
Biomonitoring	Harris et al. 2007
	Mason et al. 2005
Human health	Clarkson et al. 2003
	Grandjean et al. 2005
	Stern 2005
	Clarkson and Magos 2006
	Mergler et al. 2007

**Table 2:** Hg concentrations in subsurface water at different stations of the world ocean (range or mean ± SD).

Location	Total Hg (pM)	MeHg (pM)	Percent MeHg	References
South and Equatorial Atlantic	0.8–2.4	0.025–0.200	5–10	Mason and Sullivan 1999
North Atlantic	2.4 ± 1.6	0.029–0.160	2–7	Mason et al. 1998
North Pacific	1.2 ± 0.9	< 0.050	< 4	Laurier et al. 2004
Equatorial Pacific	0.5–4.0	0.035–0.670	2–15	Mason and Fitzgerald 1993
Mediterranean	0.5–4.0	0.020–0.460	1–35	Cossa et al. 1997, Horvat et al. 2003, Cossa and Coquery 2005

MeHg represents methylated Hg, including MeHg and dimethylmercury. Modified from Mason and Gill (2005) with permission from the Mineralogical Association of Canada.
